# Diurnal difference in CAR mRNA expression

**DOI:** 10.1186/1478-1336-2-6

**Published:** 2004-08-28

**Authors:** Yuichiro Kanno, Satoshi Otsuka, Takuya Hiromasa, Takayuki Nakahama, Yoshio Inouye

**Affiliations:** 1 Faculty of Pharmaceutical Sciences, Toho University, 2-2-1 Miyama, Funabashi, Chiba 274-8510, Japan

## Abstract

**Background:**

The constitutive androstane receptor (CAR, NR1I3) plays a key role in the transcriptional activation of genes that encode xenobiotic/steroid and drug metabolizing enzymes.

**Results:**

The expression of CAR mRNA throughout the circadian rhythm is reported for the first time in phase with the clock gene Bmal1 and in antiphase with the clock-controlled gene Rev-erbα mRNAs, with a peak at Zeitgeber time (ZT) 20 and a trough at ZT8, and a peak/trough ratio of 2.0. The diurnal difference in CAR mRNA expression might underlie the 1.7-fold difference in the magnitude of the PB-dependent induction of CYP2B1/2 mRNA.

**Conclusion:**

The circadian oscillation of xenosensor gene CAR mRNA expression is partially responsible for chronopharmacokinetics and chronopharmacology in disease.

## Background

The superfamily of nuclear hormone receptor comprises a group of transcription factors that play significant roles in response to a number of biological regulators. In addition to the pregnane X receptor (PXR, NR1I2), the constitutive androstane receptor [1] (CAR, NR1I3) plays a role in the transcriptional activation of genes that encode xenobiotic/steroid and drug metabolizing enzymes, such as cytochrome P450 (CYP) 2Bs, 2C19, 3As, multidrug resistance-associated protein 2 (MRP2), UDP-glucuronosyltransferase (UGT1A1), and 5-aminolevlinic acid synthase 1 (ALAS1) [2-8]. In response to xenobiotic PB, and other PB-like ligands such as 1,4-bis [2-(3,5-dichlorpyridyloxy)]benzene (TCPOBOP) in rodents [9] and 6-(4-Chlorpphenyl)imidazo [2,1-b][1,3]thiazole-5-carbaldehyde *O*-(3,4-dichlorobenzyl)oxime (CITCO) in humans [10], high doses of acetaminophen [11], and bilirubin [12], CAR forms a heterodimer with retinoid X receptor alpha (RXRα) and subsequently binds to the direct repeat (DR-4) motifs in such as the phenobarbital (PB)-responsive enhancer module (PBREM) in the far upstream promoter regions of mouse, rat and human CYP2B genes. In contrast, androstanol and androstenol were initially identified as inverse agonists [13] that reverse the constitutive transactivating potency of CAR.

Recently, the mRNA expression of nuclear receptors, such as peroxisome proliferater-activated receptor alpha (PPARα), retinoic acid receptor (RAR)-related orphan receptor (ROR) and RER-ERBα, have been reported to show circadian rhythms in the liver [14-17]. Hepatic PPARα mRNA and protein levels follow a diurnal rhythm which parallels that of circulating corticosterone. In addition, REV-ERBα expression is regulated by a circadian positive feedback loop attributable to the function of BMAL1/CLOCK heterodimers, and is negatively controlled by circadian negative lobe PER/CRY heterodimers [18,19].

Circadian variations in the chronopharmacokinetics and chronopharmacology of various drugs such as theophylline and propranolol have been recently reported [20]. Furthermore, daily fluctuations in hepatic P450 monooxygenase activities responsible for the first phase of metabolism of various xenobiotics are well known. For example, Cyp2a4, Cyp2a5, CYP7, and CYP3A are among those that show circadian rhythmicities [21-23] that result from the preceding rhythmic oscillations of transcription factors including nuclear receptors.

We previously determined the transcriptional start site of the rat *Car *gene (Kanno et al., 2003), resulting in the discovery of the putative REV-ERBα/ROR responsive element (RORE) at around -1.2 kb on the basis of published genomic sequence (Mazny et al., accession number AC099236). Thus, expression of the *Car *gene is expected to occur in antiphase to that of the *Per1 *gene and in phase with the *Bmal1 *gene. In the present study, the expression profile of the *Car *gene in rat liver was studied in comparison with those of the clock gene *Bmal1*, clock-controlled gene *Rev-erbα *and CAR-dependent PB-inducible *CYP2B1/2 *gene.

## Results

### Rat hepatic expression of nuclear receptor CAR mRNA follows a circadian rhythm

**Figure 1:**
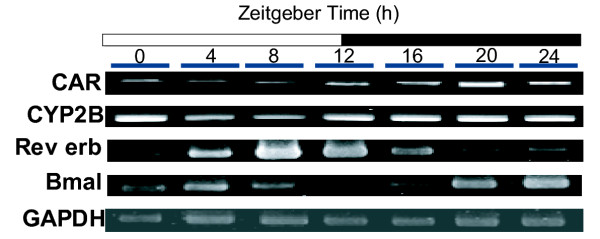
**Diurnal variations in CAR mRNA in the rat liver. **Animals were sacrificed every 4 hours at Zeitgeber times (ZT) 4, 8, 12, 16, 20 and 24/0. mRNA levels of CAR, CYP2B1/2, BMAL1, REV-ERBα and GAPDH were amplified by semi-quantitative RT-PCR. After oligo(dT)-primed cDNA was synthesized from rat liver total RNA, PCR was conducted with an initial enzyme activation step at 95°C for 5 min followed by divergent cycles of denaturation at 95°C for 15 sec, annealing at 60°C for 30 sec and extension at 72°C for 60 sec; CAR (27 cycles), CYP2B1/2, REV-ERBα and BMAL1(30 cycles), and GAPDH (24 cycles). The reaction products were separated by agarose gel electrophoresis and stained with ethidium bromide.

**Figure 2:**
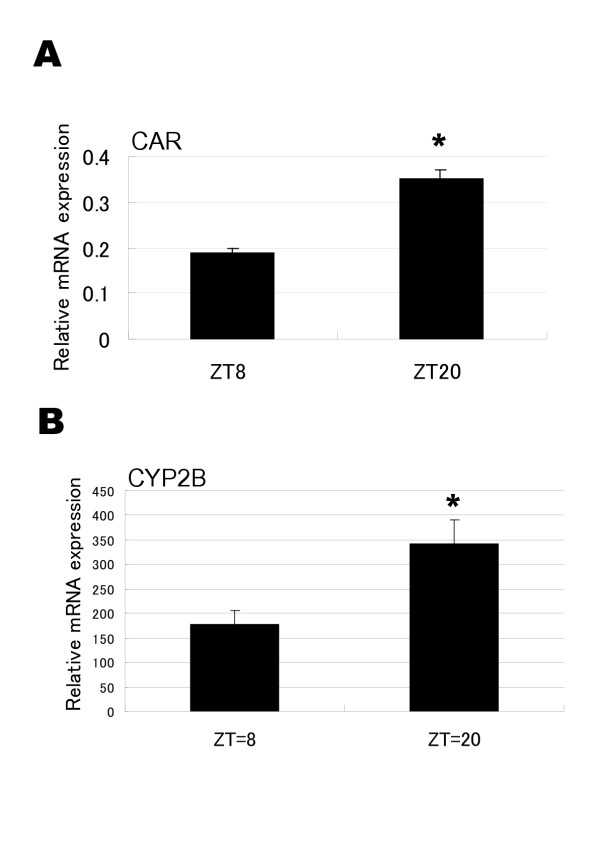
**Diurnal difference in CAR and CYP2B mRNA levels in the rat liver. **Animals were sacrificed at ZT8 and ZT20 (n = 3–4), and CAR (**A**) and CYP2B (**B**) mRNA levels were measured by semi-quantitative RT-PCR as described in the legend to Fig. 1. The results were normalized against those for GAPDH. The columns and bars represent the means ± SD with a significant difference at *: *p *< 0.01

Apart from the clock gene BMAL1 and clock-directed gene REV-ERBα, a time-dependent profile of CAR mRNA expression was observed for the rat liver. CAR mRNA levels oscillated during the day in phase with BMAL1 and in antiphase with REV-ERBα mRNAs, with a peak at ZT20 and a trough at ZT8, and a peak/trough ratio of 2.0 (Figs. 1, 2A). The CYP2B mRNA expression profile was resembled to the circadian oscillation of CAR mRNA but in a much more blunted manner (Fig. 2B).

### Diurnal difference in the induction of CYP2B by phenobarbital

**Figure 3:**
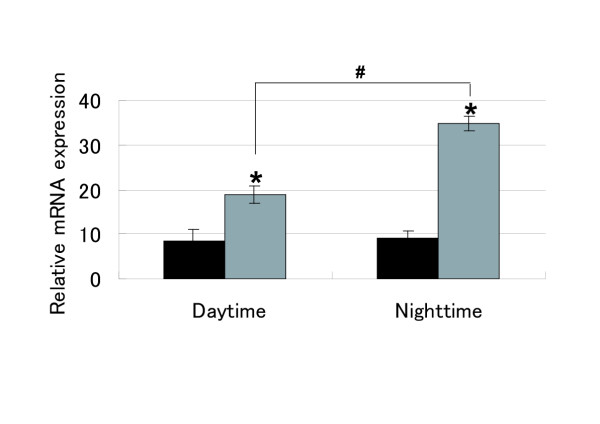
**Diurnal difference of CYP2B induction. **Animals were sacrificed at ZT13 and ZT25/1 5-hour after the injection of PB (gray columns) or veihcle (black columns) during ZT8-13 [Day] and ZT20-1 [Night], respectively. Oligo(dT)-primed cDNA was synthesized from rat liver total RNA from each animal, and CYP2B mRNA levels were measured by STBR Green real-time RT-PCR. The results were normalized against those of GAPDH. The columns and bars represent the means ± SD with significant differences compared to the individual controls at *, ^#^: *p *< 0.05

Since CAR is associated with the induction of metabolic enzymes such as CYP2B, CYP3A, and UGT1A1, the circadian rhythmicity of CAR mRNA expression may be reflected in the diurnal-difference of PB-induction of CYP2B1/2 mRNA. Therefore, we investigated the time-dependent difference of the effect of PB-treatment on the induction of CYP2B1/2 mRNA. CYP2B1/2 mRNA expression was comparatively evaluated at ZT13 and ZT1 after 5-hours of PB treatment during ZT8 to ZT13 (the minimum zone of CAR mRNA expression) and ZT20 to ZT1 (during which the expression of CAR mRNA was maximal), respectively. Hepatic CYP2B1/2 mRNA was induced 2.2-fold over the control level in the rats treated with PB between ZT8 and ZT13 [daytime treatment]. In contrast, it was increased by 3.8-fold of the control level when the rats were treated from ZT20 to ZT1 [nighttime treatment] (Fig. 3). These data suggest that the diurnal-difference in CYP2B1/2-induction might be affected by the circadian rhythm of CAR mRNA expression.

## Discussion

We previously reported that the induction of rat CYP2B1/2 by PB is absent in the lung in contrast to the marked response in the liver due to the improper splicing of CAR mRNA during its maturation. [24,25]. The longitudinal expression of CAR mRNA along the gastrointestinal tract increases from the duodenum to the terminal jejunum and then decreases toward the distal ileum while only marginal expression can be observed in the stomach and colon, implying a role for endogenous ligands such as bilirubin glucuronides secreted in the duodenum [26]. A single transcriptional start site was determined by comparison between the full-length mRNA and genomic sequences. In the present study, we investigated whether the expression of hepatic CAR mRNA shows circadian rhythmicity, because clock-controlled regulation is expected due to the presence of putative RORE in the promoter region and electrophoretic gel mobility-shift assay showed a slowly migrating band binding to the RORE probe using nuclear proteins (data not shown).

The CAR mRNA level was found oscillation daily with a peak at ZT20 and a nadir at ZT8. In contrast, BMAL1 mRNA peaked at ZT24/0 and hit the bottom at ZT12 with a 4-hours retardation, and REV-ERBα mRNA showed a peak at ZT8 and a trough at ZT20 exactly in antiphase with CAR mRNA (Fig. 1).

In contrast to the self-sustained central clock present in the brain, peripheral circadian clocks are retrained by humonal factors such as glucocorticoid hormones [27,28], as reflected in the diurnal rhythms observed for PPARα and REV-ERBα.

Glucocorticoids are also responsible for the induction of human CAR mRNA and protein via a distal glucocorticoid response element in the 5'-franking region of the gene [29], and the same might be true for its rat counterpart, which was inducible by dexamethasone (data not shown). PPARα mRNA levels followed a similar diurnal rhythm to that of the plasma level of corticosterone, which is low in the morning (around ZT2), and increases in the afternoon to reach a peak 2–3 hours before the lights out (ZT9.5). Therefore, CAR mRNA oscillation might not be retrained by the physiological diurnal variation of glucocorticoids in rats.

Recently, bilirubin was reported to be an endogenous activator of the CAR gene, which is in turn associated with the induction of bilirubin metabolising proteins, such as organic anion transporter SLC21A6, glutathione-S-transferase (GST), UGT1A1 and MRP2. Blood-bilirubin level reaches a minimum at the end of the light period and a maximum at the end of the dark period [30]. It is probable that blood bilirubin may contribute to the retraining of CAR expression to optimise bilirubin clearance.

Hepatic CYP2B1/2 mRNA level was found to be synchronized with the CAR mRNA oscillation (Fig. 1). In the clock-controlled gene cascade or network, the circadian rhythm of CYP2B1/2 mRNA expression might be partially, if not fully, explained by the hepatic CAR level. Furukawa et al. showed that hepatic P450-dependent monooxygenase activities measured by the *O*-dealkylation of 7-alkoxycoumarin fluctuate daily in F344 rats with high values during the dark period [31]. In addition, these fluctuations are regulated by a central clock present in the suprachiasmatic nucleus [32]. Further, cholesterol 7-α hydroxylase (CYP7), coumarin 7-α hydroxylase (Cyp2a4) and coumarin 15-α hydroxylase (Cyp2a5) exhibit circadian rhythmicities. These enzymes are transcriptionally regulated by albumin D-site-binding protein (DBP), which is another primary clock-controlled gene expressed according to a robust daily rhythm in the SCN and several peripheral tissues. Besides DBP, REV-ERBα is transactivated by the binding of the BMAL1-CLOCK heterodimer to the E-box motif in its enhancer region [33], and is down-regulated by the clock gene PER-CRY heterodimer. Neuronal PAS domain protein 2 (NPAS2) is highly related in primary amino acid sequence to CLOCK, being able to dimerize with BMAL1 as in the case of CLOCK. Furthermore, BMAL1-NPAS2 heterodimer was found to transactivate the same target genes as those of BMAL1-CLOCK such as *Per1*, *Per2, Cry1 and Rev-erb*α. Recently, the transcription of *Alas1 *gene encoding for the aminolevulinate synthase 1 (*Alas1*) that is rate-limitting enzyme in a heme biosynthesis was reported to be controlled in the circadian clock mechanism.

Although *Alas1 *is regulated transcriptionally by CAR-modulators having DR4 motifs in the promoter region as well as *CYP2B1/2*, BMAL1-NPAS2 and BMAL1-CLOCK heterodimers would be responsible for the daily physiological fluctuation in phase with *Rev-erbα *[34]. The circadian transcription of CAR and CYP2B1/2 is likely directly, indirectly or in combination dominated by these peripheral clocks and clock-controlled genes. The direct role of RevErb in the regulation of CAR will have to be established in further studies. For example ChIP analysis would be required to show diurnal occupancy of the putative RORE in the CAR promoter, and it has not yet been shown that Rev Erb α can modulate the transcription of the CAR promoter.

We were also interested in whether the PB-dependent induction of CYP2B1/2 mRNA is affected by the diurnal rhythm of CAR. As shown in Fig. 3, PB-treatment at night [ZT20-1] was 1.7-fold more effective than treatment during the daytime [ZT8-13] in terms of the induction of CYP2B1/2 mRNA. Although the timing of the injection of PB and monitoring of CYP2B1/2 mRNA levels adopted in this work might not have been optimal, the results obtained suggested that the diurnal difference in the expression of xenosensor genes may underlie chronopharmacokinetics and chronopharmacology in a clinical setting.

## Conclusions

Nuclear receptor CAR mRNA expression oscillates during the day with a peak at ZT20 and trough at ZT8 in antiphase with REV-ERBα, as expected due to the presence of putative ROREs in the promoter region.

Since the magnitude of PB-induction of CYP2B1/2 mRNA showed at least a 1.7-fold difference during the day, the diurnal-difference of CYP2B-induction by PB might be controlled by the circadian rhythm of CAR mRNA expression.

## Methods

### Animals and treatments

Eight week-old male Wistar rats (Clea) were kept under a 12-hours light-dark (LD12:12) cycle and provided food and water *ad libitum*. After more than 2 weeks of housing, the rats were killed at Zeitgeber times (ZT) 0, 4, 8, 12, 16, 20 and 24: ZT0 was lights-on and ZT12 is lights-out. For the PB-induction of CYP2B, the rats were i.p. injected with PB at ZT8 and ZT20 and sacrificed at ZT13 and ZT1, respectively. The livers were then dissected and used for the isolation of total RNA.

### RNA analysis by RT-PCR and Real-Time RT-PCR

Total RNA was extracted from rat liver homogenate using an RNeasy Kit (QIAGEN, Hilden, Germany). After incubation at 65°C for 10 min, the extracts were quickly placed in an ice-cold water bath. Oligo-dT primed cDNA was synthesized from 1 μg of total RNA using RTG You-Prime First-Strand Beads (Amersham Biosciences, NJ), and left at room temperature for 1 min. Reverse transcription was then performed at 37°C for 1 hour to obtain cDNA. PCR was next performed in a total reaction mixture (25 μl) containing 1 μl each of RT-reaction mixture, Ex Taq DNA polymerase (Takara, Japan) and each of primer pair. cDNA was amplified for 24 (GAPDH), 27 (CAR) or 30 (BMAL1, REV-ERVα, CYP2B) cycles of denaturation at 95°C for 15 sec, annealing at 60°C for 30 sec, and extension at 72°C for 1 min in a thermal cycler. The reaction products were separated by agarose gel electrophoresis and analyzed by a Flour Imager (Amersham Biosciences) after staining with ethidium bromide. Real-time PCR was carried out for the quantitation of each transcript in a reaction mixture consisting of 2 μl of the cDNA, 1 μl each pair of primers, 21 μl of water and 25 μl of iQ SYBER™ Green Supermix (BIO-RAD, CA). PCR was performed with an initial enzyme activation step at 95°C for 5 min followed by 50 cycles of denaturation at 95°C for 30 sec, annealing at 56°C for 30 sec and extension at 72°C for 45 sec in a real-time DNA thermal cycler (iCycler™, BIO-RAD). The following oligonucleotides were used as forward and reverse primers, respectively: 5'-ACCAGTTTGTGCAGTTCAGG-3' and 5'-CTTGAGAAGGGAGATCTGGT-3' for CAR, 5'-GAGTTCTTCTCTGGGTTGCTG-3' and 5'-ACTGTGGGTCATGGAGAGCTG-3' for CYP2B1/2, 5'-AACATGGCACTGAGCAGGTCTCC-3' and 5'-GGCATGTCCTATGAACATGTACC-3' for REV-ERBα, 5'-GCAAACTACAAGCCAACATTTCTAT-3' and 5'-CTTAACTTTGGCAATATCTTTTGGA-3' for BMAL1, and 5'-ACCACAGTCCATGCCATCAC-3' and 5'-TCCACCACCCTGTTGCTGTA-3' for glyceraldehyde-3-phosphate dehydrogenase (GAPDH). The amplified cDNA was quantitated by the number of cycles (or cross point) at which the fluorescence signal was greater than a defined threshold during the logarithmic phase of amplification. The results were shown relatively to the control level after normalization to that of GAPDH.

## Competing interests

None declared.

## Authors' contributions

K.Y. conceived of the study, carried out all experiments and drafted the manuscript. S.O. and T.H. contributed to the experiment, and N.T. participated in the design of the study and its coordination. Y.I. participated in the design of the study and drafted the manuscript in collaboration with K.Y. All authors read and approved the final manuscript.
